# Morgan and Mikhail’s Clinical Anesthesiology, 7th Edition

**Published:** 2025-07

**Authors:** John F. Butterworth, David C. Mackey and John D. Wasnick.

**Pages:** 1442 Price: $114/- **ISBN:** 978-1260473797. **Published by:** McGraw Hill / Medical, 2022

The seventh edition of Clinical Anesthesiology by Morgan and Mikhail has been available since 2022. We have procured this text book for our department at Shaukat Khanum Memorial Cancer Hospital and Research Center in Peshawar, as we believe that it will serve as a valuable resource not only for experienced clinicians but also for the junior team members and trainees. Although the history of anesthesia is commonly included in many anesthesia related texts, I was pleased to observe the inclusion of the scope of services covered in the first chapter, which was not present when I read this book as a trainee 20 years back.

A key message from the operating room environment chapter is the importance of surgical fire prevention, underscored by a flow diagram and case discussion; this often-neglected risk in the developing countries suggest a need for annual surgical fires prevention drills in anesthesia and surgical departments. In the chapter addressing cardiovascular monitoring, the inclusion of ultrasound-guided arterial cannulation would have been beneficial. In terms of the respiratory monitoring, a deeper understanding of “loops” is essential, as it is rigorously assessed during Fellowship examinations, necessitating supplementary study materials for trainees. Furthermore, a modified WHO safety checklist is mandatory for all patients receiving anesthesia and monitoring outside the operation theater. Including these details is crucial, as trainees in resource-limited countries may not have access to conventional “textbook-style” practices, particularly in settings like Pakistan. The chapter on airway management has comprehensively addressed the essential aspects of this critical skill set, including the difficult airway algorithm, the use of ultrasound, and the role of video laryngoscopes, along with other relevant airway management equipment. It is recommended that all anesthesia departments maintain dedicated difficult airway trolleys within the operating room (OR) suites, which should undergo routine checks to ensure readiness. Furthermore, there is a pressing need to establish structured training programs aimed at equipping young physicians with the necessary expertise in airway management.

Cardiovascular anesthesia should also have covered commonly seen arrhythmias in clinical practice. Core principles of pulmonary physiology are comprehensively covered within the respiratory physiology section, from which a substantial number of multiple-choice questions (MCQs) are commonly drawn. Although guidelines are provided at the end of certain chapters, it is essential to note here that this is an American publication, and some of these guidelines may not be accepted or applicable in the United Kingdom and other countries. In my view, incorporating multiple-choice questions at the end of each chapter would have been more beneficial, particularly for trainees.

Anesthetic implications of patients with neurological and neuromuscular disorders are well covered. However, for individuals preparing for their clinical component of Fellowship examinations, it is essential to have substantial practical experience with these cases. This includes managing patients with conditions such as Gullain-Barre syndrome and myasthenia gravis, as these may frequently present as long cases in examination settings. Although the renal physiology, etiology, and diagnostic criteria of acute kidney injury, along with its differential diagnosis and abdominal compartment syndrome, are thoroughly discussed, the various modalities of renal replacement therapy (RRT) are notably absent. This is an important gap, as anesthesiologists are often directly involved in the decision-making process regarding the initiation of RRT in the intensive care settings.

In summary, Clinical Anesthesiology, Seventh Edition continues to uphold its reputation as an authoritative text in the field. While a few areas might benefit from additional coverage, the text offers a well-rounded and up-to-date resource that is particularly valuable for clinicians and trainees in settings like Pakistan.

